# MRI Contrast Agents for Pharmacological Research

**DOI:** 10.3389/fphar.2015.00290

**Published:** 2015-12-09

**Authors:** Enzo Terreno, Silvio Aime

**Affiliations:** Department of Molecular Biotechnology and Health Sciences, Molecular and Preclinical Imaging Centers, University of TorinoTorino, Italy

**Keywords:** molecular imaging, MRI, contrast agents, theranosis, pharmacology

## Introduction

The advent of the molecular imaging era has offered to pharmacologists very powerful tools for drug discovery and development, *in vivo* evaluation of pharmacokinetic properties, and monitoring drug efficacy (Hargreaves, 2008; Nairne et al., 2015). In fact, molecular imaging technologies provide minimally invasive procedures to visualize, characterize, and quantify biological processes occurring at cellular/subcellular level (Weissleder and Mahmood, 2001), thus overcoming the poor clinical translatability often exhibited by *in vitro*/*ex-vivo* experimental models.

The continuous advances in biomedical imaging technologies may significantly boost the development of novel and more effective drugs, and accelerating the selection of lead compounds, with important time and costs benefits for healthcare.

*In vivo* imaging of drug delivery and release, as well as monitoring of the therapeutic outcomes, represent the base of personalized medicine, thus allowing patients to be successfully addressed to the more effective therapeutic regime.

Overall, the use of molecular imaging procedures aimed at supporting any therapeutic intervention (including surgery) falls within the scopes of theranosis (Lammers et al., 2011).

Focusing on pharmacological therapies, a typical theranostic procedure requires the design of an imaging-traceable agent, whose structure and properties are suitably tailored to the aims of the examination. Imaging drug-delivery allows the assessment of the accumulation of the drug at the biological target, thus helping the selection of the more appropriate treatment. To get accurate information, the imaging agent should have the same physico-chemical properties of the drug. This requirement can be successfully met by labeling pharmaceuticals (organic molecules, peptides, proteins, radiochelates) with PET- or SPECT-traceable radioisotopes, because of the minimal structural perturbation caused by the introduction of commonly used radionuclides (e.g., ^18^F, ^11^C, ^123^I, ^68^Ga, ^111^In; Baum et al., 2010; Gains et al., 2011; Gomes et al., 2011; Witzig et al., 2013; Wynendaele et al., 2014).

On the other hand, when the drug is loaded into a nanocarrier, also the other available imaging modalities (CT, MRI, NIRF, US, PAI) can be used to visualize the delivery of the pharmaceutical. The imaging probe can be loaded in the carrier alone or together with the drug. The first option is preferable for drug selection, the second one for monitoring therapies. Among the imaging technologies, MRI is an excellent choice because combines exquisite spatial resolution, no limits in tissue penetration, and a vast portfolio of probes and contrast modalities that allows the design/selection of the best agent for any theranostic application.

## Classification of MRI contrast agents

**Figure 1:**
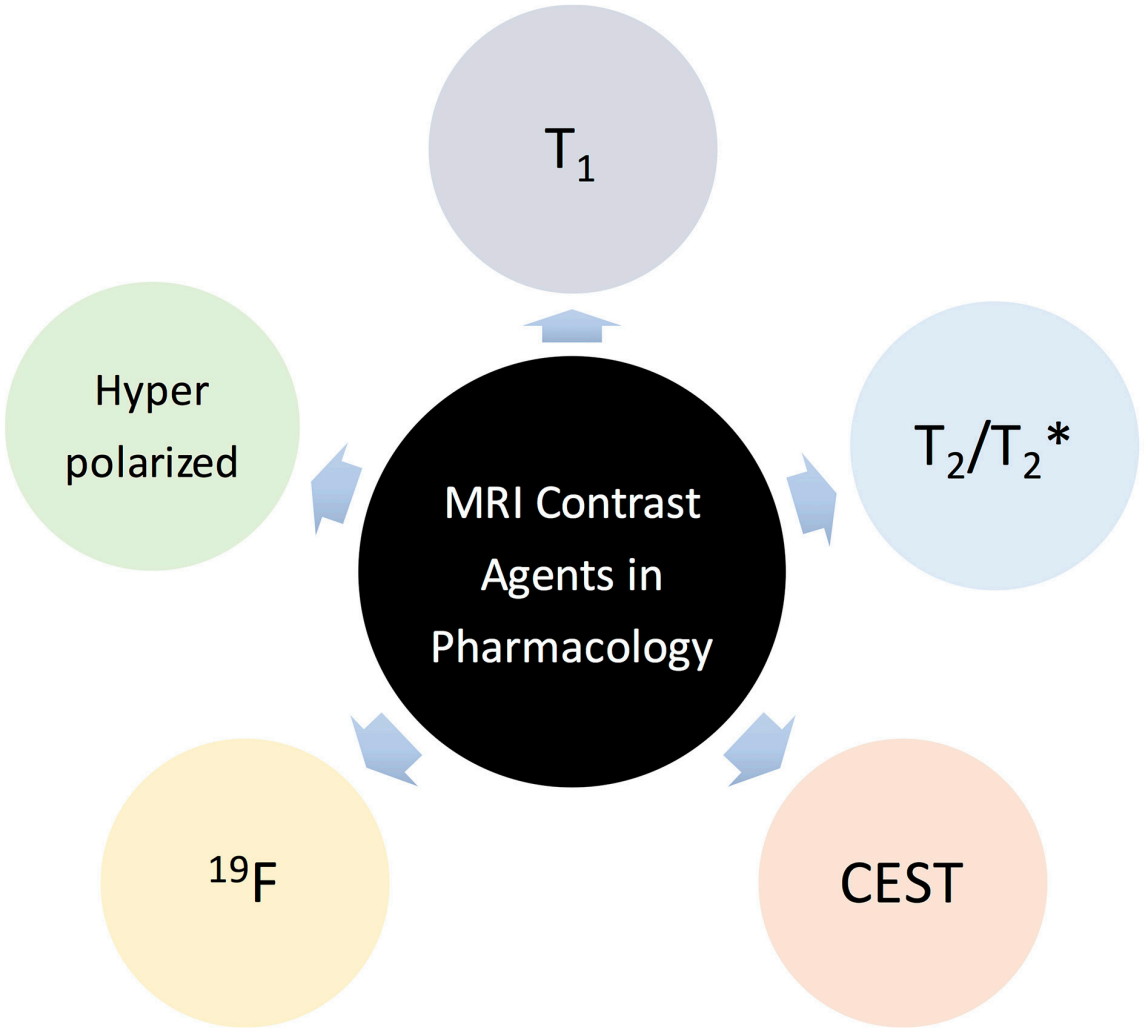
**Main classes of MRI Contrast Agents in pharmacological research**.

MRI contrast agents can be grouped in five classes: T_1_ agents, T_2_/T_2_^*^ agents, CEST agents, ^19^F-based agents, and hyperpolarized probes (Figure 1).

This section intends to provide the reader a brief description of these systems focusing on their main pro and cons with particular reference to the applications in pharmacological research.

## T_1_ agents

T_1_ agents are mostly represented by paramagnetic metal [Gd(III) or Mn(II)] complexes that enhance the MR water signal (signal brightening) in T_1*w*_ scans. The main benefits using T_1_ agents relies on the high versatility of this contrast mechanism that is affected by a large number of factors related to either structural and dynamic characteristics of the agent or biological aspects like the intra-voxel distribution of the probe (e.g., intra/extra-vascular, intra/extra-cellular). A representative example is the use of paramagnetic complexes to visualize the delivery and the release of a drug from liposomes (Viglianti et al., 2006; Hijnen et al., 2014; Rizzitelli et al., 2015). However, such systems displayed a limited sensitivity that makes necessary a local concentration of agent around 10 μM. This drawback can be partially overcome using nanometric materials that can aggregate even millions of contrastographic units, thus reducing significantly the local concentration of contrast agent (here represented by the nanosystem) necessary to generate a detectable T_1_ contrast.

## T_2_/*T*_2_^*^ agents

T_2_/*T*_2_^*^ agents are chemicals, mostly superparamagnetic nanoparticles made of iron oxides, capable to shorten the T_2_/*T*_2_^*^ of water protons much more than T_1_. Thus, their presence in the MRI image is signaled by a signal loss (darkening). Such nanoparticles show an intrinsic higher sensitivity than T_1_ agents that justifies their extensive use in MR-molecular imaging procedures, especially for cellular imaging (Srivastava et al., 2015). On the other side, these agents are often considered not to be the candidates of choice for designing smart agents, due to the difficulty to modulate the T_2_/*T*_2_^*^ contrast as a function of the microenvironment characteristics. Furthermore, the signal loss is not desirable when the target site has an intrinsically low signal (e.g., lungs, hemorrhages). However, interesting and promising theranostic applications of iron oxide nanoparticles for the visualization of drug delivery and release have been recently published (Krol et al., 2013; Liu et al., 2013).

## CEST agents

The family of CEST agents is constantly growing, and though there are no agents in clinical trials yet, the peculiarities of these systems could open new and interesting future perspectives for MRI agents in pharmacology research. The acronym CEST stands for Chemical Exchange Saturation Transfer and identifies those chemicals that generate a MRI contrast through the transfer, mediated by chemical exchange, of saturated (i.e., irradiated with a frequency specific RF pulse) protons from the donor pool (CEST agent) to the acceptor pool (bulk water).

The most important advantage of using CEST agents is that the contrast can be detected only following the irradiation of the specific NMR resonance of the donor pool. It follows that the detection of the agent is frequency-encoded and this property can be exploited for multiplex imaging or for the design of concentration-independent smart agents, both tasks being very challenging in the case of the above described relaxation agents.

The sensitivity of CEST contrast detection was recognized as an issue since the early days of the development of these agents (Ward et al., 2000). Few mM of the donor pool are necessary. However, in analogy with T_1_ agents, a large sensitivity gain can be accomplished by recurring to nanosystems. As far as the use of CEST agents in pharmacology, excellent results have been obtained using liposomes as carriers of a huge amount of exchanging water protons (the water molecules entrapped in the nanovesicles) properly shifted by the entrapment of a paramagnetic shift reagent in the inner aqueous compartment. The resulting systems (called LipoCEST, Aime et al., 2005) have been demonstrated to be very promising for imaging drug release at preclinical level (Langereis et al., 2009; Delli Castelli et al., 2010; Castelli et al., 2014).

## Heteronuclear agents

CEST agents share the frequency-encoded contrast property with agents containing MRI detectable nuclei different from protons. Among them, two classes deserve to be mentioned here because there are compounds already approved for humans or in advanced clinical trials: ^19^F agents and hyperpolarized probes.

## ^19^F agents

^19^F nuclei are the most sensitive spins after protons, and, therefore can be detected by MRI without any enrichment. The detection sensitivity is similar to CEST agents (few mM of fluorine atoms). Consequently, ^19^F agents are almost exclusively represented by nanosystems, among which perfluorocarbon nanoparticles (PFCs) are by far the most commonly used (Jacoby et al., 2013). The important advantage of fluorinated agents over the other class of contrast media stems from the possibility to directly correlate the MR signal to the agent concentration, thus allowing the quantification of targeted biomarkers and/or drugs delivered at the site of interest (Lanza et al., 2002). A commercially available formulation of PFCs will enter soon in clinical phase 1 for labeling and *in vivo* tracking human adipose-derived stem cells for breast reconstruction [^19^F Hot Spot MRI of Human Adipose-derived Stem Cells for Breast Reconstruction (CS-1000), ID NCT02035085, source: ClinicalTrials.gov].

## Hyperpolarized probes

This class of MRI agents is by far the most sensitive one, owing to the use of polarization techniques (like dynamic nuclear polarization, DNP, laser optical pumping, para-hydrogen induced polarization) that increase dramatically (up to five order of magnitude) the population difference between the spin energy levels. These agents have some similarity with PET tracer, not only for the excellent sensitivity, but also for the decay of the signal they generate (caused by the return back to the thermal polarization) that occurs on the timescale of the T_1_ of the polarized spin. Hence, one limitation in the use of hyperpolarized probes is the signal loss over time that requires fast injection and rapid accumulation at the target site.

Hyperpolarized gases (e.g., ^3^He and ^129^Xe) are clinically used for imaging the respiratory apparatus (Liu et al., 2014), whereas a ^13^C hyperpolarized compound (^13^C pyruvate) is currently in phase 1 clinical trial as metabolic agents for prostate cancer diagnosis (University of California, 2010). Besides cancer, 13C hyperpolarized agents are under intense scrutiny in cardiovascular research (Rider and Tyler, 2013).

The use of hyperpolarized probes for imaging drug delivery is quite limited, mainly due to the time constrain. Hence, their impact in pharmacological research is primarily in monitoring therapy outcome (Laustsen et al., 2014; Park et al., 2014).

A very intriguing combination between hyperpolarized and CEST agents has been proposed using ^129^Xe-based probes. The contrast arising from these agents (dubbed Hyper-CEST) relies on the reversible binding of hyperpolarized Xe with a macrocyclic host (e.g., cryptophane, cucurbituril; Schröder et al., 2006). The large chemical shift difference between the exchanging free and host-bound species allows the generation of a CEST contrast where the presence of very small amounts of the host-bound Xe can be detected after transferring its saturation to the signal of the free gas. *In vitro* proof-of-concepts highlighting the great potential and high sensitivity of these agents has been very recently published (Kunth et al., 2015; Schnurr et al., 2015).

## Conclusions

In spite of the intrinsic limited sensitivity of NMR/MRI response, several routes have been identified to allow the use of MRI probes in pharmacological studies. The enhanced sensitivity allows to take advantage of the superb spatial and temporal resolution of the MR imaging modality. On this basis, MRI is increasing its competitiveness in the Molecular Imaging arena, allowing the design of innovative experiments that provide a detailed picture of the biological microenvironment at cellular and molecular level. Moreover, functional and molecular MRI investigations imply a level of invasiveness that is definitively low in respect to the commonly used probes for nuclear medicine.

Finally, the use of frequency-encoding agents opens new horizons as they allow the visualization of more targets in the same anatomical region, i.e., they provide the access to multicolor MR images of the kind the biomedical operators are used to deal with in the histological characterization of bioptical specimens.

## Author contributions

Both the authors contributed to: (i) the design and organization of the manuscript, (ii) the drafting and critical revising of the article, and (iii) the approval of the final version to be published.

### Conflict of interest statement

The authors declare that the research was conducted in the absence of any commercial or financial relationships that could be construed as a potential conflict of interest.
